# Surface modification and coherence in lithium niobate SAW resonators

**DOI:** 10.1038/s41598-024-57168-x

**Published:** 2024-03-20

**Authors:** Rachel G. Gruenke, Oliver A. Hitchcock, E. Alex Wollack, Christopher J. Sarabalis, Marc Jankowski, Timothy P. McKenna, Nathan R. Lee, Amir H. Safavi-Naeini

**Affiliations:** 1 Department of Applied Physics and Ginzton Laboratory, Stanford University, Stanford, CA 94305 USA; 2 AWS Center for Quantum Computing, Pasadena, CA 91106 USA; 3 Flux Photonics, Inc., Pacifica, CA 94044 USA; 4 Physics and Informatics Laboratories, NTT Research Inc., Sunnyvale, CA 94085 USA

**Keywords:** Acoustics, Physics, Quantum physics

## Abstract

Lithium niobate is a promising material for developing quantum acoustic technologies due to its strong piezoelectric effect and availability in the form of crystalline thin films of high quality. However, at radio frequencies and cryogenic temperatures, these resonators are limited by the presence of decoherence and dephasing due to two-level systems. To mitigate these losses and increase device performance, a more detailed picture of the microscopic nature of these loss channels is needed. In this study, we fabricate several lithium niobate acoustic wave resonators and apply different processing steps that modify their surfaces. These treatments include argon ion sputtering, annealing, and acid cleans. We characterize the effects of these treatments using three surface-sensitive measurements: cryogenic microwave spectroscopy measuring density and coupling of TLS to mechanics, X-ray photoelectron spectroscopy and atomic force microscopy. We learn from these studies that, surprisingly, increases of TLS density may accompany apparent improvements in the surface quality as probed by the latter two approaches. Our work outlines the importance that surfaces and fabrication techniques play in altering acoustic resonator coherence, and suggests gaps in our understanding as well as approaches to address them.

## Introduction

Mechanical resonators oscillating at radio frequencies (RF) hold promise as components capable of performing important memory, processing, and transduction functions in emerging quantum systems^1–11^. However, like their superconducting counterparts, these RF acoustic systems are subject to two-level system (TLS) loss at low temperatures^7,12–14^, which limits their lifetime and utility. The standard tunneling model for two-level systems describes the temperature-dependent loss, frequency shift, and saturation behaviors^15–17^ accurately, albeit without a precise microscopic description. It is therefore unclear how different materials and fabrication approaches affect the two-level systems in a material, and their effect on the cryogenic mechanical properties.

Lithium Niobate (LN) is a popular platform for telecommunication^18^, photonics^19^, and micro-electromechanical^20^ applications as it exhibits strong photoelastic, eletro-optic, photorefractive, acousto-optic and pryoelectric effects^21^. For quantum acoustics, LN is a promising material platform due to its strong piezoelectricity^4,8,22^. However, material defects may limit how lossless devices in LN can be^23^. Thus, to use LN effectively, we must better understand what structurally dominates the losses in LN. This study systematically explores the effect of several typical acoustic resonator fabrication steps on the density of TLS in LN. We use a series of fabricated surface acoustic wave resonators (SAWs) on Lithium Niobate to study surface TLS sources in RF mechanical resonators. We choose SAWs as opposed to other types of acoustic waves because the driven mechanical motion overlaps strongly with the surface of the substrate, making them a suitable probe for surface-lying TLS sources. Moreover, they are easy to fabricate with very few processing steps, as compared to other acoustic resonators. A single metalization step on bulk LN is enough to realize a resonator, significantly reducing excess processing that could alter and complicate the final TLS density. We fabricate several resonators with different surface preparation steps and measure their TLS density at cryogenic temperatures. To develop a better understanding of the surface chemical composition, we use x-ray photoelectron spectroscopy (XPS). We also use atomic force microscopy (AFM) to investigate surface topography changes. Through this process, we identify surface treatments that increase TLS density, such as annealing or ion milling, and other surface treatments that do not alter the TLS density, such as piranha dipping, and correlate these changes with modifications of the surface properties.

## Fabrication and surface treatment

**Figure 1:**
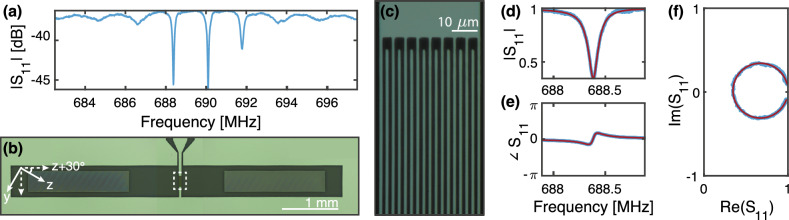
*Overview of the SAW devices* (**a**) The magnitude of $S_{11}$ across the full stopband of the device taken at 10 mK. Modes are centered at 690 MHz with a 1.7 MHz FSR. (**b**), (**c**) Optical microscope images of the SAW features: image (**b**) details the full SAW device with central IDT and Bragg mirrors on either side, image (**c**) zooms into the IDT fingers. (**d**), (**f**) Zoomed in reflection measurement (blue) and fit to a Lorentzian (red) of the first SAW mode centered at 688.4 MHz after normalization. Magnitude (**d**), phase (**e**) and real vs. imaginary parts **(f)** of $S_{11}$ are plotted for the same mode. Fit to the mode finds $Q_i = 6.8\times 10^3$ and $Q_e = 1.4\times 10^4$.

To understand the changes in the TLS-induced loss, we fabricate several SAW resonators on bulk LN, introducing varying surface treatment steps between substrate preparation and metalization. We start with a 500 µm thick bulk x-cut LN substrate from Precision Micro Optics (PMO). All but one device use congruently grown LN substrates; the other uses a 5% Magnesium Oxide (MgO) co-doped LN substrate. We prepare substrate pieces by dicing the vendor material and sonicating each piece in first acetone and then isopropanol.

Once the substrates pieces are individually solvent cleaned, we perform different surface treatments on each substrate. The “CLN” and “MgO” devices undergo no additional surface treatment- the former serving as our control group and the latter testing the effects of LN stoichiometry on TLS density. We anneal the “Annealed” devices at 500 ^∘^C for eight hours in an ambient environment. We dip the “BOE” devices in 6:1 concentration buffered oxide etchant (BOE) for 2 min following a 500 ^∘^C anneal. We submerge “Piranha” devices in a 3:1 sulfuric acid:hydrogen peroxide concentration solution for 20-min, broken into two 10 min sections. The piranha includes at 30s 6:1 BOE termination after each section. Finally, the “GCIB” devices feature no additional surface treatment until after metalization.

Once we complete surface treatments of each type, we define the SAW fingers, terminals and ground planes using photolithography and liftoff on some of the treated substrates of each type. We spin a bilayer of LOR5A and SPR3612 photoresists and expose our features using a Heidelberg MLA 150. We develop in MF26A and then deposit 25 nm thick aluminum in our Plassys evaporator. We finish the SAW metalization by lifting off in NMP solvent. The “GCIB” devices undergo a final three-minute 10 kV 30 nA ion sputter from a Gas Cluster Ion Beam to study effects of organics removal after metalization. A summary of all the surface treatment steps for each device can be found in Table 1. We then package the final SAW devices for cryogenic testing in PCBs and copper enclosures. Surface treated substrates that did not undergo metalization are reserved for XPS and AFM measurements.

**Table 1:** Surface treatment steps for each device.

Device	Treatment steps
CLN	None (Control) + metalization
Annealed	500 ^∘^C anneal, 8h + metalization
BOE	500 ^∘^C anneal, 8h + 2 min BOE dip + metalization
MgO	5% MgO co-doped substrate + metalization
Piranha	2 $\times$ (10 min Piranha + 30 s BOE) + metalization
GCIB	Metalization + 3 min 10kV GCIB

The SAW devices we pattern on all substrates consist of an inter-digitated transducer (IDT) and two Bragg mirrors, defining an acoustic cavity. The IDT consists of 17 alternating 200 µm long and 1 µm wide fingers, with a 40% duty cycle. The Bragg mirrors have 700 400-µm-long and 1.5-µm-wide fingers, with a 60% duty cycle. These parameters are chosen to optimize our stopband and free spectral range (FSR), yielding three well separated SAW modes for TLS characterization. To minimize the effect of diffraction loss caused by beamsteering in anisotropic LN and improve the internal quality factors^24^, the IDT fingers are patterned such that the SAW drive direction is parallel to the crystal Z+30^∘^ of LN, as seen in Fig. 1. We discuss more details on this aspect of the design in the supplementary material.

## Cryogenic characterization and TLS measurement

We characterize our SAW devices at low temperature, extracting the internal and external quality factors, the resonance frequencies and the loss due to TLS. To perform these measurements, we package and mount the devices on the base plate of a Bluefors LD400 dilution refrigerator, where they may be cooled to a base temperature below 10 mK. We measure temperature with a thermistor mounted adjacent to the device packaging. The setup includes a total of $-66$ dB of RF attenuation in the fridge distributed between the four temperature stages to remove most of the thermal photons in the band of interest at the device input. To obtain the reflection signal, we place two cryogenic circulators directly before the device, send in a microwave signal and measure the reflected signal on the reflection port; the reflected signal is amplified with a 4K high-electron-mobility transistor and room temperature low-noise amplifier. We fit the resulting spectra to obtain the resonance frequency as well as the intrinsic and extrinsic quality factors for all modes in our SAW stopband. At 10 mK, our SAW design yields a nominal device with a 5 MHz stopband centered at 690 MHz. The mirrors are separated by 1.75 mm giving the SAW modes an FSR of 1.7 MHz; thus, we can resolve around three SAW modes per device. At high power ($\langle n \rangle \approx 10^6$), we find that the SAW modes’ internal and external quality factors across different surface-treated substrates were within the range of $2\times 10^3< Q_i < 12\times 10^3$ and $1\times 10^4< Q_e < 3\times 10^4$. All modes are undercoupled.

To quantify the impact of TLS, we extract the average TLS loss via temperature swept spectroscopy. A signature of low-temperature TLS loss is temperature dependent resonant frequency redshifts^7,15,25^. The average loss can be expressed as the quantity $F \delta _{\text {TLS}}^0$. Here, $F$ denotes the filling fraction of TLS in the mode volume, which indicates the proportion of the volume occupied by the TLS. $\delta _{\text {TLS}}^0$ is the average TLS loss tangent, a measure of energy dissipation in the system due to the TLS. The larger the value of $F \delta _{\text {TLS}}^0$, the greater the participation of TLS loss channels. The resonance frequency of the device shifts with temperature, following this model:

1 $$\begin{aligned} \!\!\!\!\!\frac{\Delta \omega _r}{\omega _r} = \frac{F\delta _{\text {TLS}}^0}{\pi }\! \left[ \text {Re}\!\left\{ \!\Psi \!\left( \frac{1}{2}\!+\!\frac{\hbar \omega _r}{2\pi i k_B T}\right) \!\right\} \!-\!\ln \frac{\hbar \omega _{r}}{2\pi k_B T}\right] .\!\!\!\!\!\! \end{aligned}$$

Here, $\Delta \omega _r$ is the frequency shift of the mechanical oscillator from its nominal frequency $\omega _r$ at 200 mK, and *T* is the temperature. We sweep the SAW temperature from 10 mK to 200 mK using a resistive heater placed at the same temperature stage as the device; we apply the heat constantly and allow the devices to equilibrate before measurement. At each temperature, we fit the resonance frequency. As the SAW temperature decreases from the reference temperature to the base temperature, the resonance frequency of each mode decreases. For each device, we fit the temperature-dependent resonance frequency shifts to the model in Eq. 1.

**Figure 2:**
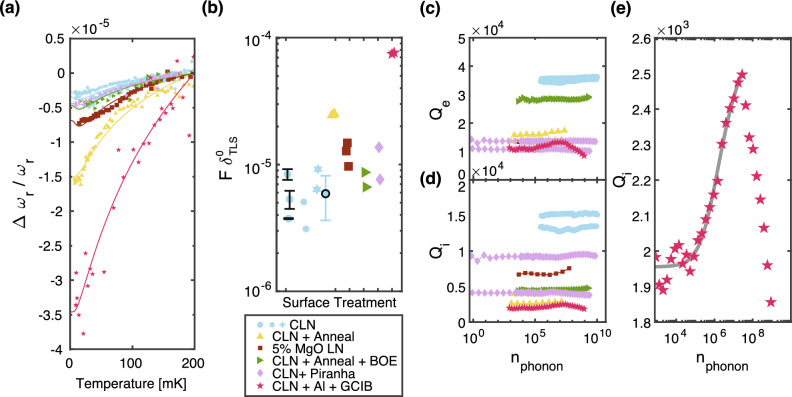
*SAW TLS measurements* (**a**) The decreased resonant frequency of different SAW modes, and each data set’s fit to Eq. 1. The modes of SAW devices that were argon ion milled with GCIB prior to cooldown had the largest redshift. The devices that had been annealed at 500 ^∘^C for 8hr at atmospheric pressure had the next largest redshift, followed by the 5% MgO co-doped device. All other surface treatment steps yielded similar low temperature redshift. (**b**) Fit $F \delta ^0_\text {TLS}$ products compared across various surface treatments. Three CLN data sets are plotted in blue. Error bars shown on the first CLN device are defined by measuring a single device multiple times per cooldown and recording changes in the redshift fit. A summary of the mean loss and error from three separately fabricated CLN devices is plotted in the black-outlined blue circle. (**c**) External and (**d**) internal quality factors are plotted as a function of mean phonon number for each of the surface processed SAW devices. A zoomed in image of the internal quality factor for the GCIB device is shown in figure (**e**). The internal quality factors are fit to Eq. 2 up to an average phonon number of $2.8\times 10^7$, plotted in gray. Beyond that, the $Q_i$ decreases once again, likely due to heating or other nonlinearities.

We compare the fit scaling factor $F \delta _{\text {TLS}}^0$ for modes on each surface treated SAW^26^, as shown in Fig. 2. The average CLN $F \delta ^0_\text {TLS}$ is $6 \times 10^{-6} \pm 2 \times 10^{-6}$, which we determine from measuring three separately fabricated control devices. The addition of GCIB sputtering yields the largest $F \delta ^0_\text {TLS}$ at $7.6\times 10^{-5}$, 13 times larger than the control CLN $F \delta ^0_\text {TLS}$. Annealing in atmosphere without a subsequent BOE dip increases the $F \delta ^0_\text {TLS}$ significantly as compared to CLN TLS loss; these modes have a TLS loss participation fraction $\approx 4$ times larger than the CLN modes. 5% MgO co-doped devices have a slightly elevated TLS loss participation, $\approx 2$ times larger than CLN. All other types of surface preparation devices have average TLS $F \delta ^0_\text {TLS}$ similar to CLN within the error bar bounds.

TLS losses can also be identified through power saturation. As power is increased within the IDT, resonant TLS will saturate and cause the linewidth of the device to decrease, described by the following equation^26^:

2 $$\begin{aligned} \frac{1}{Q_{i,\text {tot}}} = \frac{F \delta ^0_\text {TLS} \tanh (\frac{\hbar \omega _r}{2 k_B T})}{\sqrt{1 + \frac{\langle n \rangle }{n_c}^\beta }} + \frac{1}{Q_{i,\text {res}}}. \end{aligned}$$

We measure all surface treated devices at base temperature at a range of powers and extract intrinsic quality factors at average phonon levels ranging from 1 to $1\times 10^{10}$, shown in Fig 2. We see that while some of our devices have an increase in $Q_i$ with power, the relative increase is small, especially for devices with the lowest fit $F \delta ^0_\text {TLS}$ via temperature sweeps. For TLS saturation to be easily resolved through swept RF power, $Q_\text {TLS}$ must dominate the losses of the acoustic mode. We calculate our devices’ quality factors from only TLS losses at low RF power from our temperature sweep fit $F \delta ^0_\text {TLS}$ using the following equation:

3 $$\begin{aligned} Q_\text {TLS} = \frac{1}{F \delta ^0_\text {TLS} \tanh {\hbar \omega / 2 k_B T}}, \end{aligned}$$

where $\omega$ is the resonance frequency at base temperature and T is base temperature at 10 mK. We see that while the total fit internal quality factor ranges between $2\times 10^3< Q_i < 12\times 10^3$, the range of $Q_\text {TLS}$ is between $1\times 10^4$ and $3\times 10^5$, an order of magnitude larger that the total $Q_i$. Thus, while the $Q_i$’s are sufficiently large to resolve the TLS loss channels in temperature sweep measurements, other losses such as diffraction or metal losses dominate the $Q_i$ of our SAW modes, making power sweep TLS saturation effects less effective for extracting TLS density. The smallest $Q_i$ we can use to detect TLS losses through temperature sweeping is set by the signal to noise ratio of the SAW mode and fit error of the resonance frequency – the resonance frequency fit error must be smaller than the total frequency redshift.

Where possible, we fit to the $Q_{i,\text {tot}}$ and extract similar $F \delta ^0_\text {TLS}$ loss to the temperature shift model. The overall change in $Q_i$ from low power to high power ranged from 3.2% in a CLN device to 30.1% in the GCIB device. These $Q_i$ increases can be fit to Eq. 2 as seen in Fig. 2e. The fit $F \delta ^0_\text {TLS}$ for this device with power sweeps is $5.66\times 10^{-4}$, 7.6 times larger than the $F \delta ^0_\text {TLS}$ fit from the same mode via resonant frequency red shift. The difference in fit $F \delta ^0_\text {TLS}$ is likely due to the fact that fit $Q_\text {res} = 2.6\times 10^3$ is an order of magnitude less than $1/F \delta ^0_\text {TLS} = 1.3\times 10^4$, making the overall $Q_{i,\text {tot}}$ fairly insensitive to TLS power saturation. Still, the fit TLS loss from $Q_i$ follow the same general trends in surface preparation: highest TLS losses are found in GCIB devices, followed by annealed. All other surface preparations have similar TLS losses.

**Figure 3:**
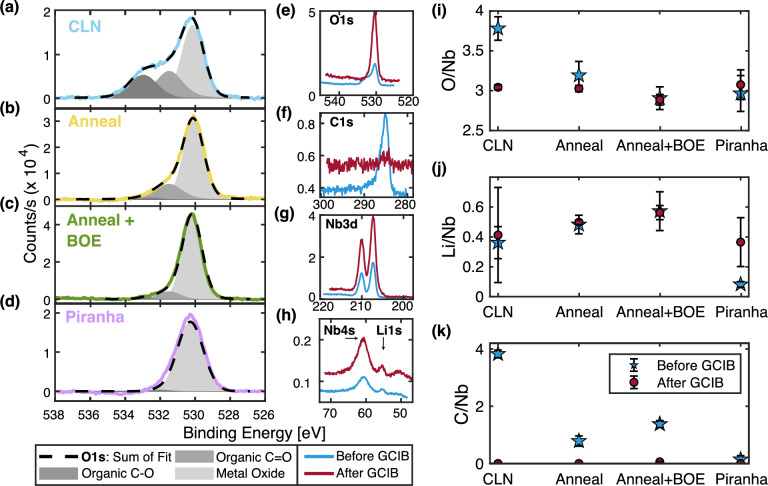
*XPS results comparing various LN surface treatments* (**a**)–(**d**) The O1*s* peak of CLN, annealed LN, annealed and BOE cleaned LN, and piranha submerged LN. Each peak is compensated from charging by shifting the measured Nb peak to LN Nb3*d*5/2 peak value of 207.3 eV; the background counts have also been subtracted from each peak. Fits to three Gaussian-Lorentzian bands for three kinds of oxygen bonding are plotted in grey: metal oxide centered at 530 eV in light grey, organic C=O bonding at 531.5 eV in medium grey, and organic C–O bonding at 533 eV in dark grey. Peaks with less of a carbon presence have negligible organic carbon peak bands, such as the case with piranha (**d**). (**e**)–(**h**) The four primary elemental peaks (O1*s*, C1*s*, Nb3*d*, and Li1*s*) of LN before (blue) and after (red) ion sputtering the surface with a Gas Cluster Ion Beam. Note that the smaller, narrower Li1*s* peak at 54.8eV overlaps with Nb4*s*, which resides at 60.2eV. Removal of surface lying organics can be seen with the near annihilation of the C1*s* peak, as well as narrowing of the O1*s* peak. (**i**)–(**k**) The relative atomic percentage ratios of each measured surface treated sample are calculated by integrating the counts in each peak. Percentages are averaged across several positions of multiple 10mm samples of each surface preparation type. Black lines represent the error bars calculated from the standard deviation between measurements of the same surface treatment.

## X-ray photoelectron spectroscopy

X-ray photoelectron spectroscopy (XPS) is a powerful analytical technique used for understanding the chemical composition of surfaces. Exploiting the photoelectric effect, an XPS measurement bombards the sample with x-rays of a known energy and identifies chemical composition of the sample from the calculated kinetic energy of emitted photoelectrons. In the context of this study, XPS provides valuable insights into the atomic and molecular constituents of the substrate surfaces, their bonding states, and how these properties change with different surface treatments. Our objective is to discern the correlation between TLS density and modifications in atomic composition of the surfaces brought about by different surface treatments. We map out the atomic composition of the surfaces by performing XPS analysis (PHI Versaprobe III XPS) of the surface of each treated substrate on “partner” chips that are not metalized. Collected photoelectrons only escape from the top 5 nm of the substrate, so this approach probes the surface chemistry changes due to surface treatment. Each XPS measurement averages a 200 $\upmu \text {m}$ diameter spot.

We also use an in-situ gas cluster ion beam (GCIB) for surface cleaning and depth profiling of samples; GCIB is advantageous for depth profiling in XPS as compared to a standard argon ion sputtering as the beam is less damaging to the sample bulk. We measure each surface-treated sample before and after an in-situ 3 min 10 kV GCIB sputter. This allows us to analyze how the surface treatments change the top 5 nm of material and verify there are no additional changes to the bulk LN stoichiometry.

We analyze the XPS spectra to obtain the average atomic percentage by summing all integrated peak counts. Each measurement collects the four strongest spectral lines for LN with surface lying contaminants: carbon C1*s*, oxygen O1*s*, niobium Nb3*d*, and lithium Li1*s*. To compensate for peak shifts due to sample charging, we shift each peak with the reference Nb3*d*5/2 peak at 207.3 eV for lithium niobate^27,28^, as it is the most prominent niobium peak of LN and it does not shift in the presence of surface organics; we choose to not shift peaks with the adventitious carbon peak reference, as this peak is removed after GCIB sputtering. We fit the total counts using a Shirley background subtraction and peak fitting (Multipak software).

The average atomic percentage for a single element *x* is given by

4 $$\begin{aligned} C_{x} = \frac{F_x^{-1}\displaystyle \int I_x \, dBE}{ \displaystyle \sum _i F_{i}^{-1} \displaystyle \int I_{i} \, dBE \, }, \end{aligned}$$

where $\int I_i dBE$ is the integrated counts of the measured elemental line after Shirley background subtraction over the binding energy, and $F_i$ is the tool specific atomic sensitivity factor, which is directly proportional to the photoabsorption cross-section^29^. To compare various spectra with the quantity best representative of stoichiometry, we report the ratios of carbon, lithium and oxygen atomic percentage to the niobium percentage. A summary of XPS atomic percentage ratio results before and after GCIB sputtering for CLN, annealed, BOE, and piranha samples can be seen in Fig. 3i–k. Black error bars represent the standard deviation of atomic percentage ratios after multiple XPS measurements of each sample type; measurements are taken in several positions on multiple 10 mm substrates with the same surface treatment. The MgO and GCIB samples are not included in this comparison as these samples have extra atomic species (magnesium in MgO samples from co-doping, and aluminum in GCIB because this surface treatment occurred after metalization), meaning that the atomic percentage ratios are not easily compared to CLN substrates.

Before performing GCIB, the CLN, Anneal, and BOE samples all have excess carbon, compared to the post-GCIB samples where carbon is undetectable. The piranha treatment nearly removes all carbon contaminants; C/Nb = 0.14 for piranha, as compared to C/Nb = 3.82 for CLN and C/Nb = 0.78 for anneal. CLN and annealed samples also have excess oxygen that is removed after GCIB. CLN has the largest relative ratios of oxygen and carbon. These relative ratios decrease from CLN levels after anneal and after anneal + BOE treatments. The measured lithium content does not vary much for different surface treatments.

The atomic percentages after the in-situ GCIB shows the efficacy of GCIB in standardizing the stoichiometry across different samples after the removal of adsorbed surface layers. We find that the samples after GCIB all have very similar stoichiometry: oxygen at $67 \pm 2 \%$, niobium at $22.3 \pm 0.2 \%$, lithium at $10 \pm 2 \%$, and carbon being undetectable. Note that there is a large systematic offset from the calculated lithium percentage and expected CLN stoichiometry; the average Li/Nb is $0.46 \pm 0.09$, when CLN should have a ratio of Li/Nb lightly less than 1 (0.95)^30,31^. We believe this to be due to systematic error caused by the the small photoabsorption cross-section of Li1*s* and subsequently the low intensity of the Li1*s* spectral peak. However, variations in the Li/Nb and O/Nb ratios from sample to sample after GCIB are much smaller than before GCIB. This suggests that differences in the measured TLS densities may be due to variations in adsorbed oxygen and carbon on the top few nanometers of CLN, annealed, and BOE dipped samples, rather than by significant changes in the bulk of the crystal.

We also use XPS spectra to study chemical bonding type via peak line shape. The bonding type of surface lying oxygen can be determined from the O1*s* spectral line shape. When there is excess organic contaminant on the surface from residual resists, higher energy carbon bonding bands at 533 eV and 531.5 eV take up a larger percentage of the total O1*s* counts^32–34^. On the other hand, the primary oxygen bonding in lithium niobate is an $\text {Nb}_2\text {O}_5$ metal oxide centered at 530.5 eV. The changes in oxygen bonding bands can be seen in Fig. 3a–d. CLN control samples which have no additional cleaning after removal of dicing resist via solvents have the largest presence of C–O and C=O oxygen bonding. Piranha dipped LN samples target and remove organics, such that the organic carbon bonding bands are absent in this sample. Annealed and annealed plus BOE cleaned samples have a larger prominence of metal oxide bonding than CLN, but still show some organic oxygen bonding in the C=O shoulder.

Similarly, Fig. 3e–h show how the all the spectral lines change on a CLN sample after GCIB sputtering. The C1*s* line is almost completely removed, showing removal of surface lying organics and adventitious carbon. O1*s* line narrows as all oxygen atoms now contribute to metal oxide bonding. The line shape of the Nb3*d*, Nb4*s*, and Li1*s* peaks are unchanged.

Finally, we use the in-situ GCIB to argon ion sputter the metalized SAW device on the “GCIB” sample, which we subsequently cool down for microwave measurements. Just as in the CLN device, we see a removal of oxygen and carbon species. This sample probes the effects of removed surface organics and adventitious carbons after metal liftoff, as well as argon ion sputtering of the surface.

**Figure 4:**
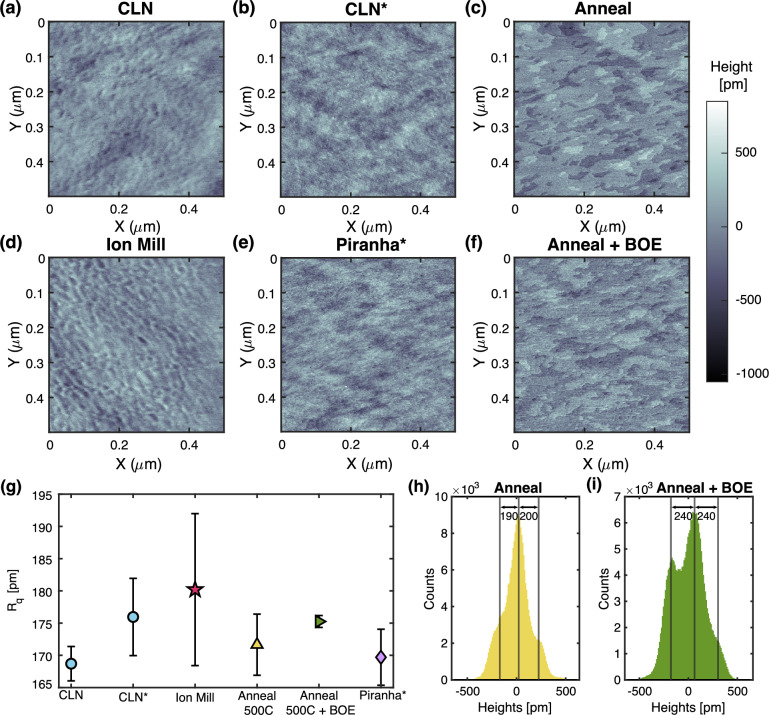
*AFM of various surface prepared LN substrates* (**a**)–(**e**) 0.5 $\upmu$m square, 512 pixel peak-force mode AFM image of unprocessed congruent LN, argon ion milled LN, annealed at 500 ^∘^ C for 8 hr LN, annealed and BOE dipped LN, and piranha dipped LN. Asterisk on the piranha sample and one CLN sample denote material sourced from a separate area of the wafer. Each image is made on x-cut material, with the X scanning direction of the AFM along crystal Z+30^∘^. A change in surface morphology is noticed after annealing, and maintained after BOE dip. (**g**) The RMS roughness of each material process type; error bars are determined from several AFM images. (**h**), (**i**) The histograms of the flattened anneal and anneal + BOE samples. The histograms are fit to a sum of three Gaussian peaks to determine the step heights between atomic terraces. Mean step height of the annealed sample is $200 \pm 80\, \text {pm}$, and $240 \pm 80\,\text {pm}$ for annealed + BOE. Uncertainty is set by the fit standard deviation of the Gaussian peaks.

## Atomic force microscopy

To further correlate our TLS findings with surface properties, we image several surface treated LN samples with atomic force microsocopy (AFM). We use a Bruker MultiMode-8 HR atomic force micrsocope in peak-force mode to image x-cut LN samples with the same surface preparation steps as the SAWs. All AFM images are taken such that the AFM scanning direction X is along crystal Z+30^∘^ of the LN. From the AFM scans we investigate how surface processing changes surface morphology and roughness between the CLN, annealed, BOE, piranha and argon ion milled samples. We remove sample tilt in each AFM scan with a 1-d polynomial background subtraction prior to image processing and analysis. We measure CLN on two separately fabricated substrates to demonstrate changes in morphology in different locations of the wafer.

Surface morphologies of the six AFM studied substrates are shown in Fig. 4a–f. The surface morphologies of the CLN, piranha, and ion milled samples are similar, exhibiting some pitting and soft height changes across the image. However, significant changes in the surface morphology are observed after the annealing process. For the annealed samples, there are groupings of higher and lower heights in isolated islands. This is likely a signature of LN surface termination relaxation from non-crystalline LN to single atomically-flat terraces of crystalline LN. We verify that there are unique step heights by further flattening these images using a three-point level on one individual atomic terrace and plot its histogram. The histograms are fit to a sum of three Gaussian peaks, where the centers of each Gaussian peak are the mean heights of each atomic layer. The mean step height of the annealed sample is $200 \pm 80~\text {pm}$, and $240 \pm 80~\text {pm}$ for annealed + BOE. Previous work using AFM to study annealed x-cut LN finds the step heights between surface terraces to be $0.24 \pm 0.2~\text {nm}$^35^, in close agreement with our findings.

**Table 2:** *Summary of the temperature sweep, XPS, and AFM results for each surface treatment.*

Surface treatment	$\overline{F \delta ^0_\text {TLS}}$	Pre-GCIB O/Nb	Pre-GCIB C/Nb	AFM surface morphology	AFM roughness $R_q$ [pm]
CLN	$5.8\times 10^{-6}$	3.78	3.83	Pitted/Soft height variations	172
Annealed	$2.48\times 10^{-5}$	3.19	0.78	Atomically-flat islands	172
BOE	$7.7\times 10^{-6}$	2.91	1.38	Atomically-flat islands	175
MgO	$1.24\times 10^{-5}$	N/A	N/A	Pitted	560
Piranha	$1.06\times 10^{-5}$	2.97	0.14	Soft height variations	170
GCIB	$7.53\times 10^{-5}$	N/A	N/A	Pitted/Soft height variations	180

Reported $F \delta ^0_\text {TLS}$ is calculated from the average of all fitted resonances of each surface type. XPS numbers reported are the averaged oxygen and carbon atomic percentage ratios before performing the in-situ GCIB sputter. AFM results are reported as both the summary of surface morphology types and roughness. MgO and GCIB devices are not compared to other samples after XPS measurement because of other atomic species present (Mg and Al, respectively).

We also calculate the RMS roughness of each sample after initial 1-d polynomial background tilt removal. Argon ion milling roughened the surface most significantly, and also increased the variability of $R_q$ across the chip, seen in Fig. 4g as larger error bars for argon ion milled devices. The addition of annealing and the BOE dip after annealing does not change the surface roughness significantly, when compared to the control sample’s RMS roughness and error bars. AFM images and roughness of the MgO sample is excluded from Fig. 4 as this material was sourced from a separate wafer (see the supplementary material for this data and analysis).

## Results and future experiments

In this investigation, we explore surface treatment methods and the impact of doping on LN to understand their effects on quantum acoustic applications. A summary of the results from all studied surface treatments can be found in Table 2. We find that both thermal annealing and GCIB processing can significantly improve aspects of the surface quality as observed by two methods: AFM and XPS. Thermal annealing results in less disordered surfaces as per AFM results, whereas GCIB processed surfaces show improved characteristics, i.e., reduced carbon and associated oxygen bonding, under XPS examination.

Contrary to our expectations, however, despite improving surface quality in certain aspects, these treatments increase two-level system (TLS) density for the samples we tested. High TLS density is detrimental to the performance of quantum systems, and significant efforts have started to illuminate the effects of fabrication processes on TLS emerging in superconducting qubits ^36–38^. For the case of lithium niobate mechanical resonators, our results seem somewhat contradictory as the surface quality improvement apparent in XPS/AFM is accompanied with increased TLS density for the GCIB and annealed samples. Furthermore, the tested BOE and piranha samples demonstrate that the surface quality improvements found in annealed and GCIB samples are not the cause of TLS loss increase as BOE and piranha have similar surface quality improvements (less disordered surfaces and adsorbed carbon and oxygen reduction, respectively) and a measured TLS density that is lower and closer to the control. Rather, the processes of argon ion milling and annealing seem to change the material in ways that XPS and AFM are not able to detect. This signifies a gap in our knowledge that must be addressed to optimize these systems’ performance.

Our results with MgO co-doped lithium niobate add to this puzzle. Congruently grown lithium niobate is nonstoichiometric as it forms from a melt consisting of only 48.6% $\text {Li}_2\text {O}$^23^; MgO co-doping fills vacancies of lithium and is an important technique used to improve LN’s optical photorefractive properties. We do not observe a reduction in TLS as was suggested by earlier experiments on phononic crystals^7^. Our findings show a slight *increase* in TLS density with MgO co-doping.

Despite the surprising questions raised by our results, they shed light on the crucial role surfaces play in the performance of quantum acoustic resonators. We can now assert with high confidence that the resonators investigated here are surface-limited. Moreover, the results of the GCIB experiment show that though the metal-LN surface may play a role, a process affecting only the LN surface significantly changes the observed TLS density. The characterizations we have performed on these devices help us identify paths for deeper investigations to unlock the full potential of LN-based quantum acoustic devices.

Future work should consider incorporating a larger number of advanced characterization techniques to unravel the complex interplay between surface treatment, TLS density, and doping. Transmission electron microscopy (TEM) provides high-resolution imaging and analysis that can help us understand changes in LN’s crystal structure near the surface due to different treatments. MgO co-doping, which did not yield the expected reduction in TLS in our experiments, could be re-evaluated with material from another vendor, akin to that from Ref.^7^. Furthermore, current fabrication methods using resist-based liftoff processes could be replaced by resist-free processes, potentially reducing the TLS density^39^. Alternatively, using a metal other than aluminum that can undergo more aggressive cleaning processes might reduce surface TLS.

Progress in quantum acoustics needs a more granular understanding of the effects of surface treatment techniques and material alterations on dissipation and dephasing. While unearthing unexpected results, our findings underscore the critical importance of surface properties in LN-based quantum acoustic resonators and point to a path forward for understanding and hopefully mitigating TLS to enable powerful quantum acoustic technologies.

### Supplementary Information

Supplementary Information.

## Data Availability

The data supporting the conclusions of this study are available from the corresponding author upon reasonable request.
