# Supplementary material for: Surface modification and coherence in lithium niobate SAW resonators
Source: Sci Rep. 2024 Mar 20;14:6663. doi: 10.1038/s41598-024-57168-x (PMC10954613; doi:10.1038/s41598-024-57168-x)
Supplement: Supplementary file 1 — Supplementary Information. [file 41598_2024_57168_MOESM1_ESM.pdf]

Supplemental information for "Surface Modification and Coherence in Lithium Niobate SAW Resonators"

1 Reducing Diffraction Loss

Due to the anisotropy of lithium niobate, SAWs fabricated on x-cut LN can have a non-zero beamsteering angle. To reduce diffraction losses in our SAW devices and therefore improve Q_{res}, we find a SAW drive direction on the LN crystal which minimizes the beamsteering angle through simulation. Using an FEM solver[1], we model an IDT unit cell on x-cut Lithium Niobate, sweeping the IDT drive direction from crystal $Z-90°$ to $Z+90°$. The beamsteering angle η for each crystal drive orientation is solved by calculating:

$$\eta = \arctan \frac{\oiint_s P_{\perp} ds/A_{\perp}}{\oiint_s P_{\parallel} ds/A_{\parallel}}, \tag{1}$$

, where $P_{\parallel}$ and $P_{\perp}$ are the power through the parallel and perpendicular faces to the SAW drive direction, respectively, and similarly $A_{\perp}$ and $A_{\parallel}$ are the areas of each designated unit cell face. We find two drive directions on an x-cut crystal that correspond to a beamsteering angle of $0°$: $Z-30°$ and $Z+75°$, the first being the crystal orientation used in this study.

Surface acoustic waves are commonly fabricated on several crystal cuts of LN including X, Y, 128Y[2]. While we use this FEM technique to find minimum beamsteering angle on x-cut LN, this procedure could also be used on other crystal cuts of LN to maximize internal quality factor of SAW resonators. We choose to use x-cut LN to easily compare fabrication related TLS density changes to previous studies on TLS density in LN[3].

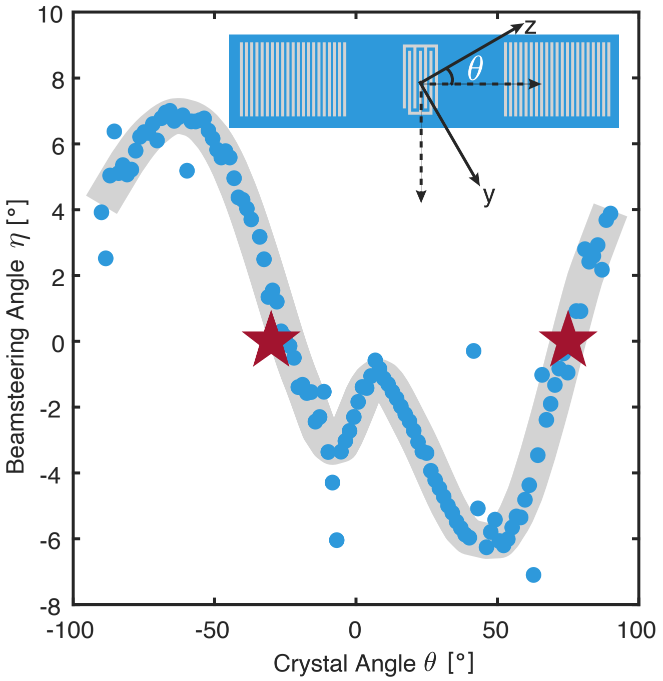

Fig. S1. A plot of x-cut Lithium Niobate beamsteering angle as a function of SAW drive crystal direction, blue is solved from FEM model, and grey is the smoothed data. The inset shows a schematic for how θ is defined, where the dotted lines represent the SAW drive direction and the solid arrows show true crystal Z and Y. The red stars represent the two identified crystal orientations with zero beamsteering angle, optimum for reducing diffraction loss on x-cut LN.

2 Dark Modes

Many of the SAW modes do not have a perfect Lorentzian line shape. We often see other more weakly coupled modes at nearby frequencies as shown in Fig S2. We associate this effect with "dark" modes that are only electromechanically coupled through

the primary SAW mode. We fit this collection of modes more accurately to a model where a dark mode is weakly coupled to the drive through the SAW mode. Modes with dark modes are fit to the function

$$S_{11} = 1 - \frac{\kappa_e}{i\Delta + \kappa/2 + \frac{g^2}{i\Delta_b + \gamma/2}}, \tag{2}$$

where Δ and Δ_b are the detunings from the drive frequency to the primary mode and dark mode, respectively, κ_e is the external coupling rate to the primary mode, κ is the total loss rate of the primary mode, γ is the internal loss rate of the dark mode, and g is the coupling rate between the primary and the dark mode.

We use this model to fit all SAW modes in our temperature sweep measurements, to more accurately extract the resonance frequency and therefore the TLS loss product.

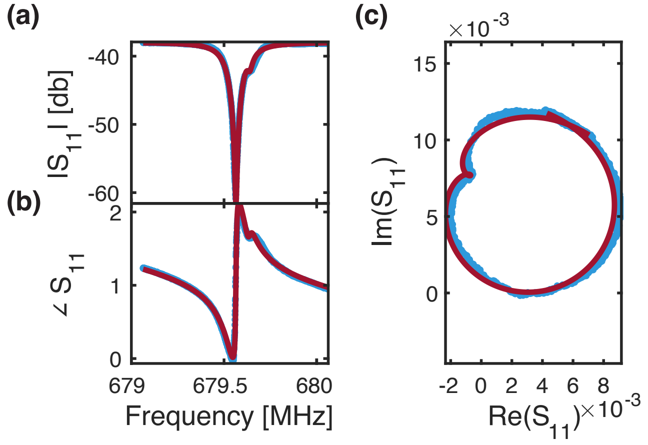

Fig. S2. *SAW Dark Mode Fit* (a) Magnitude, (b) phase and (c) imaginary vs real parts of measured (blue) and fit (red) S_{11}. This SAW resonance demonstrates a small dark mode 75kHz above the primary resonance ($\omega_{\text{dark}} = 2\pi \times$ 679.639 MHz). This mode came from the BOE device. The data is fit to equation 2.

3 AFM of MgO

We AFM image our vendor supplied 5% MgO co-doped wafer sourced from PMO, shown in Fig S3. The wafer was supplied with different polishing constraints, such that the mean roughness is much larger than our other CLN surface treated samples. The wafer similarly shows pitting consistent with polished material that has not been annealed. The mean roughness of this material is 560 ± 60pm.

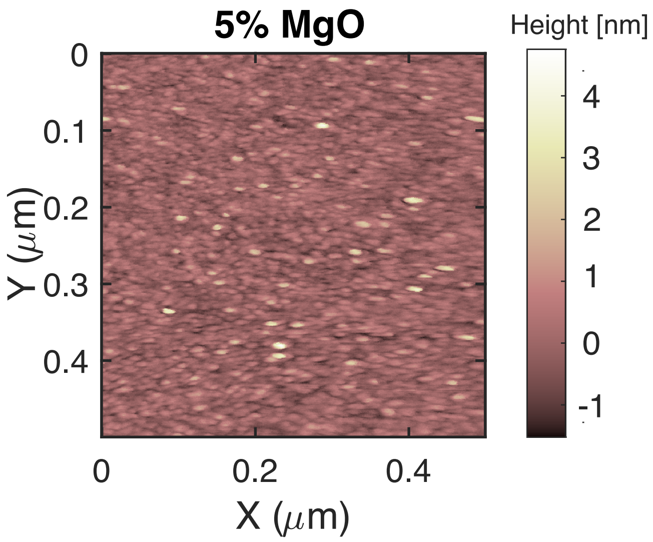

Fig. S3. *5% MgO co-doped LN AFM*

References

1. COMSOL Multiphysics v. 5.4. https://www.comsol.com.

2. Dneprovski, V. G., Karapetyan, G. Y. & Parinov, I. A. *Surface acoustic wave devices* (Nova Science Publishers, Inc., 2016).

3. Wollack, E. A. *et al.* Loss channels affecting lithium niobate phononic crystal resonators at cryogenic temperature. *Appl. Phys. Lett.* **118**, 123501 (2021).
